# GnRH Regulates Sex Differentiation in *Sebastes schlegelii* Through TGF-β/MAPK Signaling Pathways

**DOI:** 10.3390/biology15110857

**Published:** 2026-05-30

**Authors:** Jinwei Huang, Pingrui Xu, Yongshuang Xiao, Jun Li

**Affiliations:** 1 Key Laboratory of Breeding Biotechnology and Sustainable Aquaculture, Institute of Oceanology, Chinese Academy of Sciences, Qingdao 266000, China; huangjinwei@qdio.ac.cn (J.H.); xupingrui2024@163.com (P.X.); 2 Laboratory for Marine Biology and Biotechnology, Qingdao Marine Science and Technology Center, Qingdao 266237, China; 3 University of Chinese Academy of Sciences, Beijing 100049, China; 4 College of Life Sciences, Qingdao Agricultural University, Qingdao 266109, China; 5 Shandong Province Key Laboratory of Experimental Marine Biology, Institute of Oceanology, Chinese Academy of Sciences, Qingdao 266071, China

**Keywords:** *Sebastes schlegelii*, GnRH, sex differentiation

## Abstract

Sex determination and gonadal differentiation are essential for vertebrate development and reproduction. Fish, with both genetic and environmental sex-determining mechanisms, serve as ideal models for exploring environmental effects on gonadal development. The hypothalamic–pituitary–gonadal (HPG) axis is involved in sex differentiation, but its role as a key mediator in temperature-dependent sex differentiation remains unclear. *Sebastes schlegelii* is an economically important marine fish species along the northern coast of China. It exhibits obvious sexual growth dimorphism and typical temperature-dependent sex differentiation, making it an excellent model for investigating the mechanism of sex differentiation. In this study, individuals of *S. schlegelii* at the sex differentiation stage were treated with a GnRH receptor antagonist and subjected to transcriptome sequencing analysis. The results revealed that GnRH may regulate sex differentiation through steroid biosynthesis, TGF-β and MAPK signaling pathways. We identified several key genes, including *srd5*, *bmp8a*, *bmp2*, *fgf23*, *pdgfra*, and *egfr*, which may affect gonadal differentiation by acting on core sex-determining genes and steroidogenesis. In conclusion, GnRH precisely mediates the process of sex differentiation by modulating the TGF-β signaling pathway, the MAPK signaling pathway, and local gonadal steroidogenic networks.

## Simple Summary

Sex determination and gonadal differentiation are critical for fish growth and development. Unlike humans, fish sex is influenced by both genes and environmental factors such as water temperature. *Sebastes schlegelii*, an economically important marine fish in northern China, exhibits sex-related growth performance, and water temperature can affect offspring sex ratio. Our previous study found that gonadotropin-releasing hormone (GnRH) is involved in high-temperature-induced sex differentiation, but the mechanism remains unknown. Using transcriptome analysis, we discovered that GnRH regulates sex differentiation through three key pathways: steroid biosynthesis, TGF-β signaling, and MAPK signaling. Several key genes involved in this process were also identified. These findings have important implications. First, they help scientists understand how water temperature influences sex determination in fish, providing insights for predicting climate change impacts on fishery resources. Second, by artificially manipulating these pathways and genes, it may be possible in the future to selectively produce more females (which grow faster) in aquaculture, thereby improving economic efficiency.

## 1. Introduction

In recent decades, climate warming driven by greenhouse gas emissions has continuously reshaped the pattern of global ecosystems, and marine organisms exhibit particularly significant responses to such changes [1]. The Sixth Assessment Report of the Intergovernmental Panel on Climate Change (IPCC) indicates that global ocean heat content shows an irreversible upward trend, with the upper ocean warming rate reaching 0.11 °C per decade, accompanied by frequent extreme thermal events and intensified seasonal fluctuations [2,3]. The uneven spatiotemporal distribution and amplified variation amplitude of seawater temperature not only directly drive population decline and affect genetic diversity, but also weaken the structural stability and service supply functions of marine ecosystems through trophic cascade effects [4,5,6,7]. As key nodes in marine food webs, fish undertake the dual functions of maintaining ecological balance and supporting fishery economic output, and their life-history traits are highly sensitive to changes in thermal environments [8,9,10]. Continuous warming profoundly interferes with the growth and development of fish through multiple pathways, such as metabolic remodeling, oxidative stress activation, and immune suppression, posing a severe challenge to the long-term survival of fish populations [11,12,13,14].

Sex determination and gonadal differentiation constitute the core biological basis for development and reproduction in vertebrates [15]. Fish exhibit extremely diverse sex determination mechanisms, which are mainly classified into two categories: genotypic sex determination (GSD) and environmental sex determination (ESD). Sex differentiation in most species arises from the combined effects of genetic and environmental factors [16,17]. Among sex determination systems, chromosomal sex determination (CSD) is the most common form, mainly including the XX/XY male heterogametic model and the ZZ/ZW female heterogametic model [18,19,20]. Furthermore, the sex-determining genes of most fish are not restricted to sex chromosomes; some species even lack morphologically differentiated sex chromosomes, rendering their sex differentiation processes readily regulated by environmental factors [21,22]. Among these, temperature-dependent sex determination (TSD) is widespread in taxa such as Pleuronectiformes and Perciformes [23]. Temperature effects on sex differentiation can influence population dynamics. On the one hand, water temperature during the larval stage can modulate the direction of gonadal differentiation to a certain extent, thereby affecting the sex ratio of offspring. On the other hand, male- or female-skewed sex ratios induced by extreme thermal events may reduce reproductive capacity and threaten population sustainability [24,25]. Meanwhile, the TSD mechanism also provides a technical breakthrough for unisexual breeding in aquaculture. Precise temperature control to manipulate sex can significantly improve aquaculture production and economic benefits, serving as an important bridge linking basic research to industrial application.

*Sebastes schlegelii* is an economically important marine fish species along the northern coast of China, with an annual output of approximately 10,000 tons. This species exhibits significant sexual growth dimorphism (females grow faster than males), and previous studies have shown that it undergoes feminization at 24 °C and masculinization at 27 °C [26]. These two characteristics provide a technical entry point for monosex aquaculture and also make it an ideal model for dissecting the mechanism underlying high-temperature-mediated sex differentiation. Previous studies have mostly focused on the direct effects of sex-related gene expression and sex steroid hormone synthesis. Recent studies have indicated that the fish hypothalamic–pituitary–gonadal axis (HPG axis) participates in the process of sex differentiation [26]. Gonadotropin-releasing hormone (GnRH) serves as the core regulatory factor of the HPG axis; it stimulates the pituitary to release follicle-stimulating hormone (FSH) and luteinizing hormone (LH), thereby regulating gonadal steroidogenesis and gametogenesis [27]. Gene knockout studies in zebrafish have confirmed that the deletion of GnRH3 can lead to male-skewed sex ratios in populations, revealing for the first time that a specific GnRH isoform is involved in sex determination [28]. However, the regulatory mechanism of GnRH in sex differentiation remains unclear, especially in *S. schlegelii*, for which no relevant research has been reported.

This study aimed to elucidate the regulatory mechanism of GnRH in sex differentiation of *S. schlegelii*, filling the gap in neuroendocrine regulation research of this species. Using GnRH receptor antagonist treatment combined with transcriptome and qRT-PCR analyses, we demonstrated for the first time that GnRH signaling pathway is essential for sustaining female development. The GnRH signaling pathway regulates sex differentiation together with the key steroidogenic enzyme *srd5* via the TGF-β signaling pathway (*bmp8a*, *bmp2*) and MAPK signaling pathway (*fgf23*, *pdgfra*, *egfr*). This study not only clarifies the central role of the GnRH signaling pathway in integrating neuroendocrine and local gonadal signaling networks, but also provides theoretical basis and molecular targets for analyzing the dynamics of population sex ratio and developing monosex breeding techniques of *S. schlegelii* under climate warming.

## 2. Materials and Methods

### 2.1. Ethics Statement

All experiments were conducted in strict compliance with the experimental animal guidance policy as outlined by the Institute of Oceanology’s Animal Ethics Committee, Chinese Academy of Sciences (Permit No. IOCAS20230916PPFA0007).

### 2.2. Culture Protocol of Experimental Animals

Experimental larvae of *S. schlegelii* were obtained from Weihhai Shenghang Technology Co., Ltd. (Weihai, Shandong Province, China). On the first day after parturition, the larvae were reared in an environment of 18 °C ± 1 °C under a photoperiod of 14 h light/10 h dark. This water temperature of 18 °C ± 1 °C was maintained throughout the entire larval rearing and subsequent feeding trial period. The selection of this specific temperature range was based on two considerations. First, it falls within the suitable range for juvenile growth, as previous research has demonstrated that water temperatures of 16–20 °C are conducive for rearing this species, with optimal growth performance observed at 20 °C [29]. Second, this temperature range is representative of the ambient water temperature measured at the local aquaculture facility where the broodstock was maintained, ensuring consistency with practical production conditions. After 30 d of rearing, healthy and uniformly sized juvenile fish were randomly selected and transferred to six 140 L tanks at a density of 200 individuals per tank, with three tanks assigned as the control group and the other three as the experimental group. During the larval rearing period, a recirculating aquaculture system was used, with each tank equipped with independent water supply and aeration devices. One-third of the water volume was replaced daily with sand-filtered and aerated seawater. The experimental group was fed a diet supplemented with the GnRH antagonist Relugolix (Cat. No. HY-16474; MedChemExpress LLC, Monmouth Junction, NJ, USA) at a dosage of 3 mg/kg body weight, as recommended by the manufacturer’s instructions. qPCR was used to detect the expression of downstream genes to verify the treatment effect of Relugolix. The control group received the same basal diet. Feed was provided manually, and the feeding ration was adjusted weekly based on changes in body weight. The feeding ration was kept consistent between the control and experimental groups, and all other rearing conditions were identical except for the presence or absence of Relugolix in the diet. The experiment lasted for 90 days. At 42 days post-hatching, nine individual fish were collected from each experimental group (control group and Relugolix-treated group). Every three individuals were pooled as one biological replicate, yielding a total of three biological replicates per group for transcriptome sequencing. Fish remaining after the 42-day sampling were reared until the end of the experiment (90 days) for phenotypic sex identification. The tail of each fish was excised and frozen in liquid nitrogen for genetic sex identification. The remaining whole-fish tissues were stored separately in liquid nitrogen, Bouin’s fixative, and paraformaldehyde (PFA) fixative for other subsequent experimental analyses. Whole-fish samples were used instead of gonadal tissues because the juvenile fish used in this study were extremely small, making it impossible to isolate gonadal tissues by dissection.

### 2.3. Extraction of Genomic DNA and Total RNA, and Synthesis of cDNA

DNA was extracted for the identification of genetic sex in *S. schlegelii*, while cDNA was extracted for the detection of gene expression profiles. The TIANamp Marine Animal DNA Kit was utilized to extract DNA from fish tail tissues, while TRIzol Reagent (Invitrogen, Carlsbad, CA, USA) was employed for isolating total RNA. A NanoDrop 2000 spectrophotometer (Thermo Fisher, Waltham, MA, USA) was used to evaluate the quality and concentration of both DNA and RNA. The Evo M-MLV RT Kit (Accurate Biology, Changsha, China) facilitated the synthesis of cDNA. Both DNA and cDNA were preserved at a temperature of −20 °C.

### 2.4. PCR-Based Genetic Sex Identification

Genetic sex identification was performed following the method of Song et al. [30], DNA extracted from *S. schlegelii* tail tissues was used as a template, and PCR amplification was conducted using specific primers targeting single-stranded DNA fragments (Table S2). A total of 30 individuals were used for genetic sex identification in this experiment.

### 2.5. Transcriptome Sequencing

To explore the molecular differences and characteristics of *S. schlegelii* following Relugolix treatment, we performed transcriptome sequencing on juvenile fish from the control and experimental groups. Nine whole fish were collected in each experimental group. Every three individuals were pooled as one biological replicate, with a total of three biological replicates per group. Total RNA was extracted from each sample, reverse-transcribed into cDNA, and purified to obtain double-stranded DNA templates. After end repair, adapter ligation, and PCR amplification, sequencing libraries with an insert size of 350 bp were constructed and subjected to 150 bp paired-end sequencing on the Illumina NovaSeq 6000 platform (Gene Denovo Biotech Co., Guangzhou, China). Data quality control was conducted using FASTP (v0.20.0) to ensure clean reads. Reference genome alignment was performed using Hisat2 v2.1.0 and Bowtie2 v2.5.4, and gene expression levels were calculated with RSEM.

### 2.6. Screening, Enrichment and Expression Pattern Analysis of DEGs

To analyze the differential RNA expression between the two groups, the DESeq2 software (v1.38.3) was employed [31], with the screening criteria for differentially expressed genes (DEGs) set as a false discovery rate (FDR) < 0.05 and an absolute fold change ≥ 2. For gene expression quantification, raw read counts for each transcriptional region were calculated using the RSEM software (v1.3.3) [32] and used for subsequent differential expression analysis. Additionally, FPKM values were also calculated by RSEM for visualization of expression abundance across samples. Principal component analysis (PCA) was conducted with the R software package (v4.2.2) (http://www.r-project.org/), aiming to reveal the similarity among different biological samples based on the overall gene expression levels. Additionally, the clusterProfiler R package was utilized to perform Gene Ontology (GO) enrichment analysis and KEGG functional enrichment analysis, so as to predict the potential functions of DEGs. The threshold for significant enrichment of gene sets was defined as *p* < 0.05.

### 2.7. Gene Set Enrichment Analysis

To more comprehensively clarify the regulatory effect of specific pathways, we used gene set enrichment analysis (GSEA) software (v4.3.3) combined with the MSigDB database [33] to perform gene set enrichment analysis, and simultaneously conducted KEGG enrichment analysis to verify whether differentially expressed genes are enriched in specific pathways. After inputting the gene expression matrix, genes were sorted using the signal-to-noise ratio normalization method, and then enrichment scores and *p*-values were calculated according to the default parameters of the software. Specifically, based on the expression data of all genes, we selected the most widely used Signal2Noise as the gene sorting standard; by analyzing the specific positions of the target gene set in the sorting of all genes, we quantitatively scored the pathways and GO terms associated with the gene set, and then obtained the enrichment score (ES value). Subsequently, permutation tests were performed based on the gene set to calculate the significance *p*-value, and finally, the normalized enrichment score (NES value) and false discovery rate (FDR value) were obtained through the multiple test correction process.

### 2.8. Quantitative Real-Time PCR

To detect the mRNA expression levels of genes associated with key pathways identified through transcriptomic analysis, we used the Bio-Rad CFX96™ Real-Time Detection System (Bio-Rad, Hercules, CA, USA) and quantitative real-time polymerase chain reaction (qRT-PCR). The qRT-PCR primers were designed using the NCBI Primer-BLAST online tool (based on BLAST+ v2.15.0, http://www.ncbi.nlm.nih.gov/tools/primer-blast (accessed on 22 December 2025)). After verifying no cross-reactivity by BLAST, the primers were synthesized by Sangon Biotech (Shanghai, China) Co., Ltd. The primer sequences are detailed in Table S1. The total volume of the qRT-PCR reaction system was 20 μL, consisting of 10 μL SYBR Green I PCR Master Mix, 0.4 μL forward primer, 0.4 μL reverse primer, 7.2 μL deionized water, and 2 μL cDNA. The amplification procedure began with an initial denaturation step at 95 °C for 30 s, followed by 40 cycles of 95 °C for 5 s, gene-specific annealing temperature for 30 s, and 72 °C for 30 s for extension. Each sample had three technical replicates, and based on previous research findings, we selected *btf3l4* as the reference gene [34]. The Pfaffl method was employed to quantify mRNA expression levels [35].

### 2.9. Data Analysis

Statistical analyses were conducted using GraphPad Prism version 9.0.0. Group comparisons were assessed by one-way Analysis of Variance (ANOVA). Results are expressed as mean ± standard error (SE), with significance set at *p* < 0.05.

## 3. Results

### 3.1. Transcriptome Sequencing Assembly and Analysis

To investigate the molecular expression changes of sex differentiation-related genes in *S. schlegelii* under Relugolix treatment, genetically female individuals were firstly screened for the experiment. Genetically female *S. schlegelii* without any treatment were set as the control group, and Relugolix-exposed genetically female individuals were designated as the experimental group. Reference-based transcriptomic analysis was performed on the *S. schlegelii* transcriptome. High-throughput sequencing was performed on the Illumina NovaSeq 6000 platform, generating a total of 3.164 × 10^7^ raw reads. After quality control and trimming using FASTP (v0.20.0), a total of 3.1509 × 10^7^ clean reads were obtained. The quality of the clean reads was assessed, with Q20 and Q30 values exceeding 98.72% and 96.29%, respectively, meeting the basic quality criteria (Q20, Q30 ≥ 90%). The clean reads were then aligned to the reference genome using HISAT2 v2.1.0 software (Table 1).

### 3.2. Transcriptome Profiling and Differentially Expressed Gene Identification

**Figure 1:**
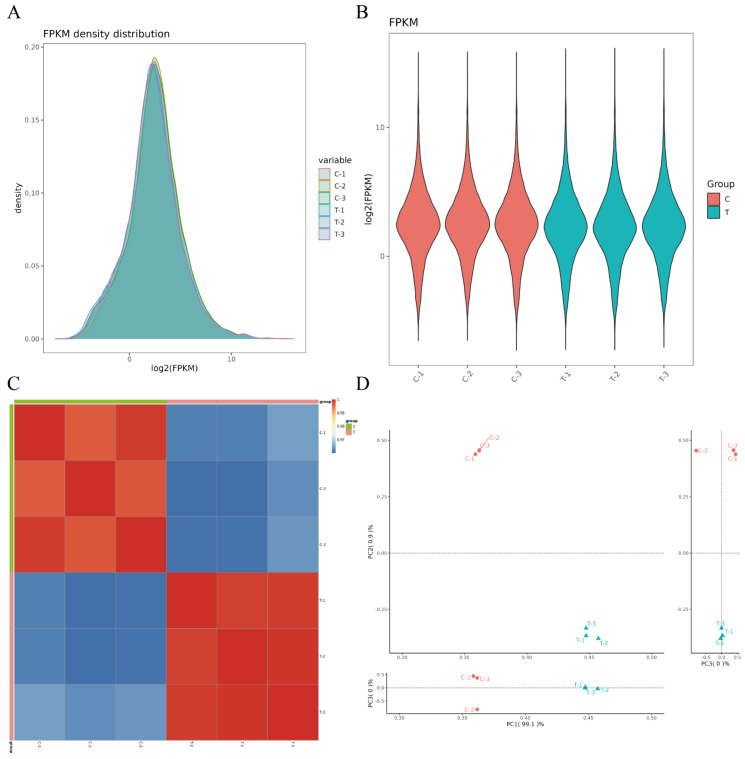
FPKM-based transcriptomic gene expression and sample correlation analysis. Note: (**A**) Distribution of gene expression abundance. (**B**) Violin plot of gene expression. (**C**) Heatmap of correlations among three different sample groups, with the depth of color representing the magnitude of the correlation coefficient between two samples. Within the same color tone, the deeper the color, the stronger the correlation. (**D**) Principal component analysis (PCA) plot.

Based on the FPKM values of each gene in the assembled transcriptome, the gene expression distribution profile showed the expression level distribution of genes across six distinct samples (Figure 1A,B). Correlation analysis of gene expression levels among samples (Figure 1C) revealed high similarity between biological replicates within the same group, indicating high experimental reliability and reasonable sample selection. Meanwhile, principal component analysis (PCA) and Pearson correlation coefficient analysis demonstrated good reproducibility of samples within each group, suggesting that the sequencing data obtained were reliable and suitable for subsequent analysis (Figure 1D).

**Figure 2:**
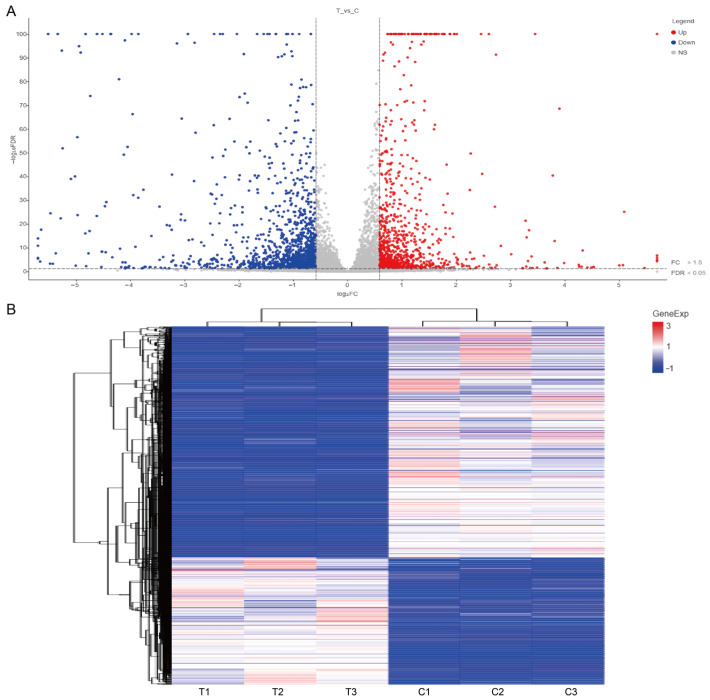
Volcano plot and cluster heatmap analysis of differentially expressed genes. Note: (**A**) Volcano plot of differentially expressed genes (DEGs) among different groups. Blue represents downregulated genes, red represents upregulated genes, and gray represents genes with no significant difference in expression. (**B**) Heatmap of cluster analysis for DEGs among different groups. The horizontal axis represents samples from different groups, and the vertical axis represents DEGs. The expression levels of DEGs in different samples are indicated by different colors: the redder the color, the higher the expression level; the bluer the color, the lower the expression level.

A total of 2538 differentially expressed genes (DEGs) were identified by differential expression analysis (screening criteria: FDR < 0.05 and |log_2_FC| > 1.5). Compared with the control group, 1047 genes were upregulated and 1491 genes were downregulated in the experimental group. The volcano plot illustrated the fold change and statistical significance of the DEGs (Figure 2A), and the clustering heatmap displayed the expression clustering of DEGs across all samples (Figure 2B).

### 3.3. GO and KEGG Enrichment Analysis of Differentially Expressed Genes

**Figure 3:**
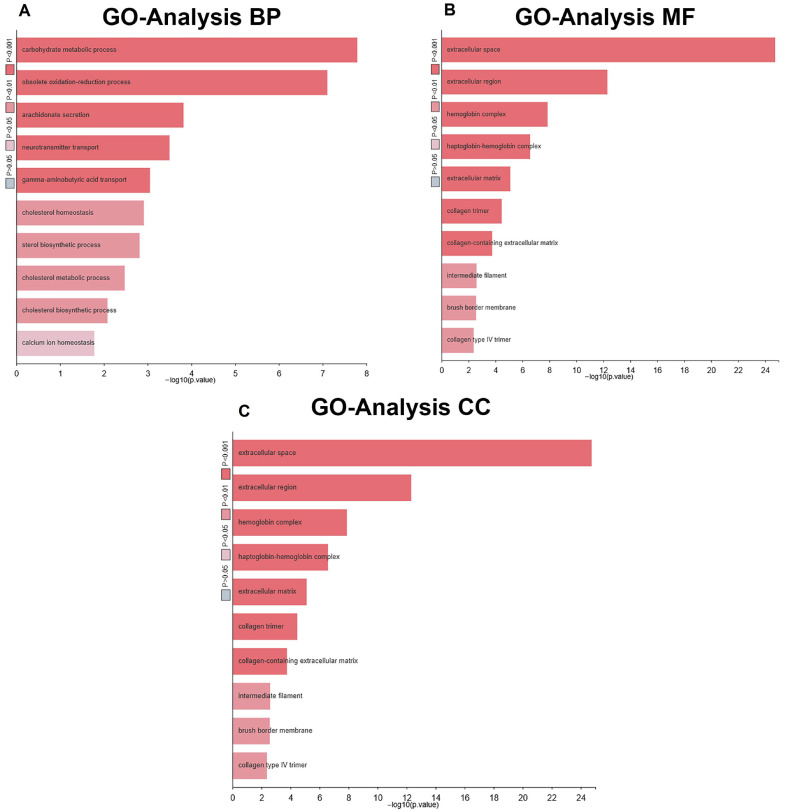
GO classification of differentially expressed genes. Note: (**A**) Enrichment analysis of biological processes of DEGs among different groups. (**B**) Enrichment analysis of molecular functions of DEGs among different groups. (**C**) Enrichment analysis of cellular components of DEGs among different groups. The horizontal axis represents the −log_10_-transformed *p* value, and the vertical axis represents the enriched functional terms. The color of the bar indicates statistical significance: the closer the color is to red, the higher the significance; the closer the color is to gray, the lower the significance.

**Figure 4:**
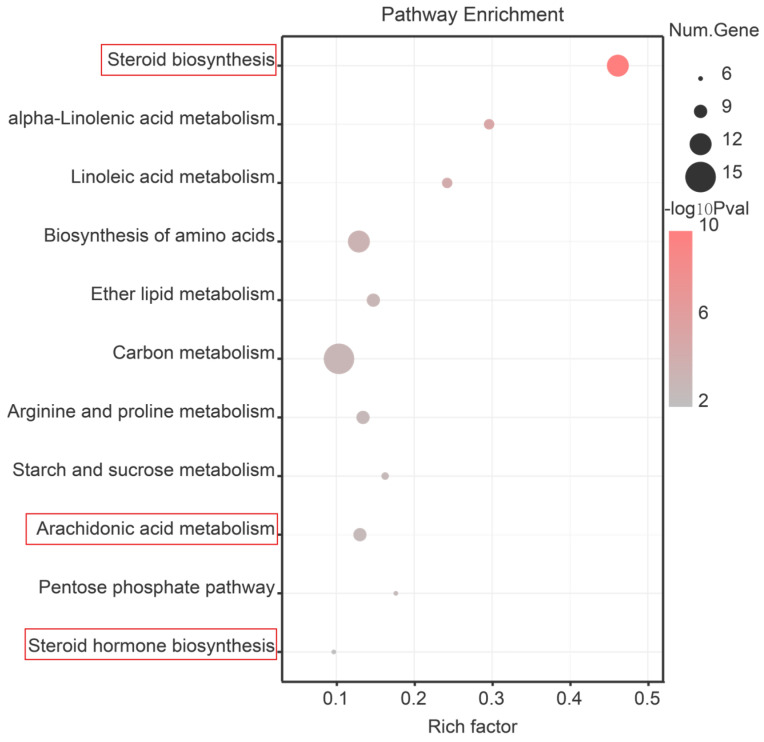
KEGG enrichment analysis of differentially expressed genes. Note: The horizontal axis represents the enrichment factor (Rich Factor), which is the ratio of the input genes enriched to the target pathway to the annotated genes. The vertical axis represents the enriched pathway entries, and the color of the bubbles indicates the statistical significance.

To identify the functional categories and pathways associated with the differentially expressed genes, GO functional annotation and KEGG pathway enrichment analysis were performed on the set of differentially expressed genes. GO analysis revealed that the differentially expressed genes were significantly enriched in all three main functional categories (Figure 3). In terms of Biological Process, pathways related to energy metabolism and lipid signaling were significantly enriched (FDR < 0.05), including carbohydrate metabolic process, oxidation–reduction process, and arachidonate secretion. In terms of Molecular Function, catalytic function terms were notably enriched, such as serine-type endopeptidase activity, serine-type peptidase activity, and hydrolase activity. In terms of Cellular Component, secretion-related compartments were enriched, including extracellular space, extracellular region, and hemoglobin complex. KEGG pathway enrichment analysis showed that the differentially expressed genes were significantly enriched in pathways closely related to sex differentiation, including steroid biosynthesis, arachidonic acid metabolism, and steroid hormone biosynthesis (Figure 4).

### 3.4. Gene Set Enrichment Analysis of All Expressed Genes

To complement the traditional enrichment analysis and identify pathway-level trends that may not be detected by differentially expressed gene-based approaches, GSEA was performed based on a ranked list of all expressed genes according to their differential expression fold change. Unlike the KEGG enrichment analysis in Section 3.3, which was based on a pre-selected set of differentially expressed genes, GSEA evaluates the coordinated behavior of entire gene sets across the whole transcriptome.

**Figure 5:**
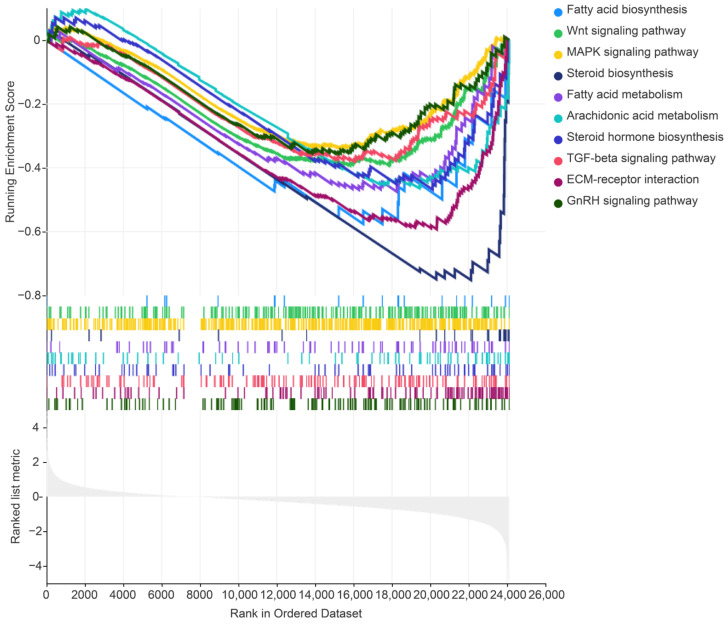
Gene set enrichment analysis. Note: The horizontal axis represents the ranking of genes. According to the gene ranking results, genes included in the KEGG entry are assigned positive scores, while those not included are assigned negative scores. The maximum value obtained from this scoring is defined as the maximum value of the entry. The positions of positive scoring are marked with vertical lines below the plot. The vertical axis denotes the cumulative enrichment score, where the peak value corresponds to the enrichment score of the gene set. The bar segment displays the distribution of ranking scores of all genes (genes are sorted in descending order of signal-to-noise ratio values).

The GSEA results revealed that gene sets related to steroid hormone regulation, including the steroid biosynthesis pathway, steroid hormone biosynthesis pathway, and arachidonic acid metabolism pathway, were significantly enriched in the Relugolix-treated group (Figure 5). In addition, gene sets associated with upstream signaling regulation, such as the Wnt signaling pathway, MAPK signaling pathway, TGFβ signaling pathway, and GnRH signaling pathway, also showed enrichment trends. Notably, some pathways (e.g., steroid biosynthesis and arachidonic acid metabolism) were enriched in both methods, while others (e.g., Wnt and GnRH signaling pathways) were detected only by GSEA, highlighting the complementary nature of the two approaches.

### 3.5. qRT-PCR Validation of Transcriptomic Data

**Figure 6:**
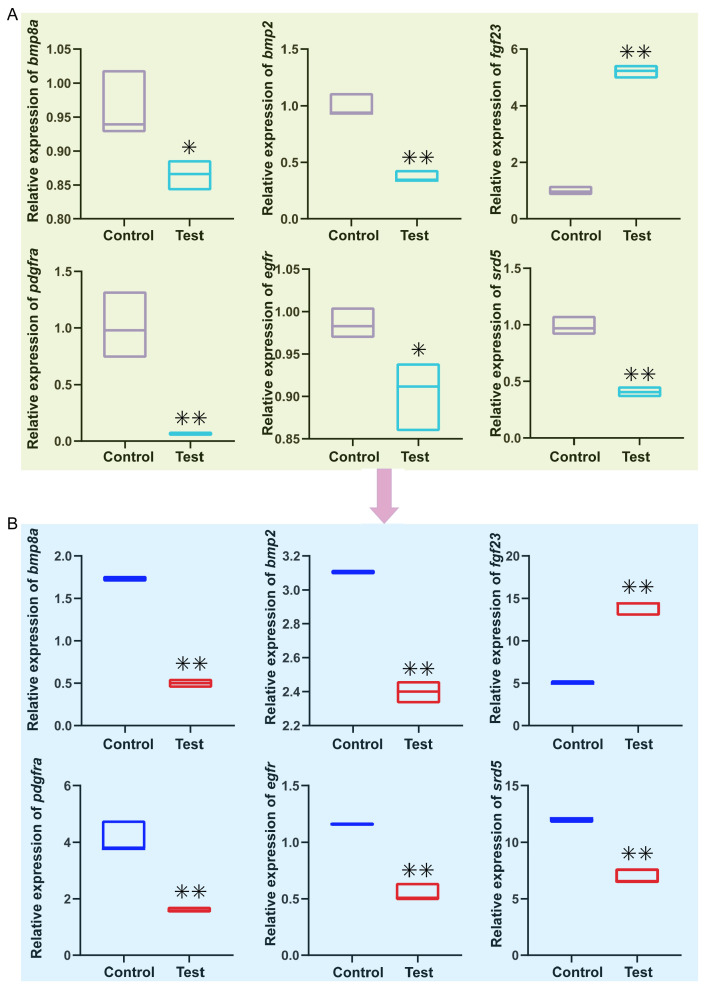
Analysis of the expression differences of target genes between the control group and the experimental group. Note: The *x*-axis represents different groups. The control group was the normal culture group at 18 °C, and the experimental group was the Relugolix-treated group at 18 °C. (**A**) Expression detected by qRT-PCR; (**B**) Expression in the transcriptome. * indicates a significant difference in expression level in the experimental group compared with the control group (*p* < 0.05), and ** indicates an extremely significant difference (*p* < 0.01).

To verify the reliability of differentially expressed genes from the transcriptomic analysis, six genes (*bmp8a*, *bmp2*, *fgf23*, *pdgfra*, *egfr*, *srd5*) involved in pathways closely related to the experimental phenotype were selected for qRT-PCR validation. The results showed that the expression patterns obtained by qRT-PCR were consistent with those from the transcriptome (Figure 6), confirming the reliability of the transcriptomic data. In addition, differentially expressed genes from the steroid biosynthesis pathway, MAPK signaling pathway, and TGF-β signaling pathway were selected for validation. It was found that in the experimental group, the expression of *bmp8a* and *bmp2* in the TGF-β signaling pathway was significantly downregulated. In the MAPK signaling pathway, *fgf23* was significantly upregulated, whereas *pdgfra* and *egfr* were significantly downregulated. The steroid synthesis-related gene *srd5* was also markedly downregulated.

## 4. Discussion

### 4.1. GnRH Is Involved in the Sex Differentiation of S. schlegelii

Comparative transcriptomic analysis was performed on gonads of genetic female *S. schlegelii* between the control group and the Relugolix-treated group. We revealed that differentially expressed genes were significantly enriched in the steroid biosynthesis pathway in the KEGG analysis, suggesting that GnRH signaling may participate in the masculinization process of genetic female *S. schlegelii* by regulating gonadal steroid hormone synthesis. Meanwhile, combined with GSEA and existing studies related to sex differentiation [28,36,37], we focused on the TGF-β signaling pathway, MAPK signaling pathway, Wnt signaling pathway, and calcium signaling pathway. Although these pathways did not show the most significant enrichment among the overall differentially expressed genes, they exhibited obvious enrichment trends in GSEA and were highly associated with vertebrate gonadal development, sex determination, and differentiation. This indirectly indicates their important potential roles in GnRH-mediated gonadal sex remodeling.

The results of GO functional enrichment analysis were generally consistent with the biological functions of the aforementioned pathways. In terms of biological processes, differentially expressed genes were mainly enriched in the carbohydrate metabolic process, oxidation–reduction process, and arachidonate secretion. Based on these observations, we speculate that Relugolix treatment may be associated with alterations in energy metabolism, redox homeostasis, and lipid signaling molecule secretion, which might provide a material and energy basis for gonadal structural remodeling and sex phenotype conversion [38,39,40]. This result is also consistent with the significant enrichment of the arachidonic acid pathway in the KEGG analysis. At the molecular function level, differentially expressed genes were enriched in serine-type endopeptidase activity, serine-type peptidase activity, and hydrolase activity. Previous studies have indicated that these enzymatic activities are closely related to extracellular matrix remodeling, growth factor maturation, and the cleavage and processing of signaling molecules [41,42,43]; accordingly, we speculate that they may play a role in regulating signal intensity and spatiotemporal distribution by influencing the activation and degradation of ligands and receptors in the TGF-β and MAPK pathways [41,42,43]. Cellular component analysis revealed that differentially expressed genes were mainly localized in the extracellular space, extracellular region, and hemoglobin complex. This distribution pattern may suggest that active extracellular signal communication, cellular microenvironment remodeling, and alterations in local oxygen supply and metabolic status occur in the gonad during sex remodeling, which is partially consistent with the extracellular signaling-dependent mode of action of the TGF-β and MAPK pathways [43,44,45].

### 4.2. Potential Mechanisms Underlying the Roles of Key Genes

Further screening and validation revealed that the key genes of the TGF-β signaling pathway, *bmp8a* and *bmp2*, were significantly downregulated after Relugolix treatment. The MAPK signaling pathway-related gene *fgf23* was significantly upregulated, while *pdgfra* and *egfr* were significantly downregulated. Meanwhile, the expression of *srd5*, a key gene involved in steroid synthesis, was markedly decreased. Taken together, we hypothesize that GnRH signaling pathway may regulate the TGF-β and MAPK signaling pathways, thereby affecting steroid hormone biosynthesis and ultimately mediating the differentiation of genetic female *S. schlegelii* toward a male phenotype. As a key member of the TGF-β superfamily, *bmp2* dynamically regulates FSH receptor expression and estradiol synthesis in ovarian granulosa cells via the Smad1/5/8 pathway, serving as a core factor maintaining folliculogenesis and granulosa cell differentiation [46]. In genetic female (XX) gonads, *bmp2* acts as a downstream effector of the Wnt4-Rspo1-β-catenin pathway and cooperates with *foxl2* to activate *fst* expression, thereby sustaining ovarian development and indirectly repressing male-related genes such as *sox9* [47,48,49].

Based on the transcriptomic data from the present study, we speculate that the significant downregulation of *bmp2* after blockade of the GnRH signaling pathway triggers a series of cascade reactions. On the one hand, the decreased expression of *fst* may affect the stability of *foxl2*, thereby weakening the function of upstream factors such as wnt4 and rspo1, and reducing β-catenin pathway activity, which might not favor the maintenance of the female differentiation pathway. On the other hand, the repression of *sox9* may be subsequently relieved, accompanied by increased expression of male-related genes such as *fgf9* and *amh*, which might, to some extent, shift the primordial gonad toward testicular development. Furthermore, previous studies have suggested that *bmp8a* may promote the proliferation of Sertoli cells in the testis [50]; its downregulation may reflect developmental timing disturbances caused by GnRH deficiency. In the present study, the significant upregulation of *fgf23* and downregulation of *pdgfra* and *egfr* suggest that GnRH signaling pathway may be associated with specific branches of the FGF-FGFR-MAPK cascade. The downregulation of *pdgfra* and *egfr* may affect the survival signals of ovarian somatic cells [26,51,52], while the upregulation of *fgf23* may influence the function of testicular interstitial cells through paracrine mechanisms [26,52]. These changes suggest that the MAPK signaling network may undergo a pro-male shift and may cooperate with the TGF-β pathway to influence gonadal development.

### 4.3. Limitations and Future Perspectives

**Figure 7:**
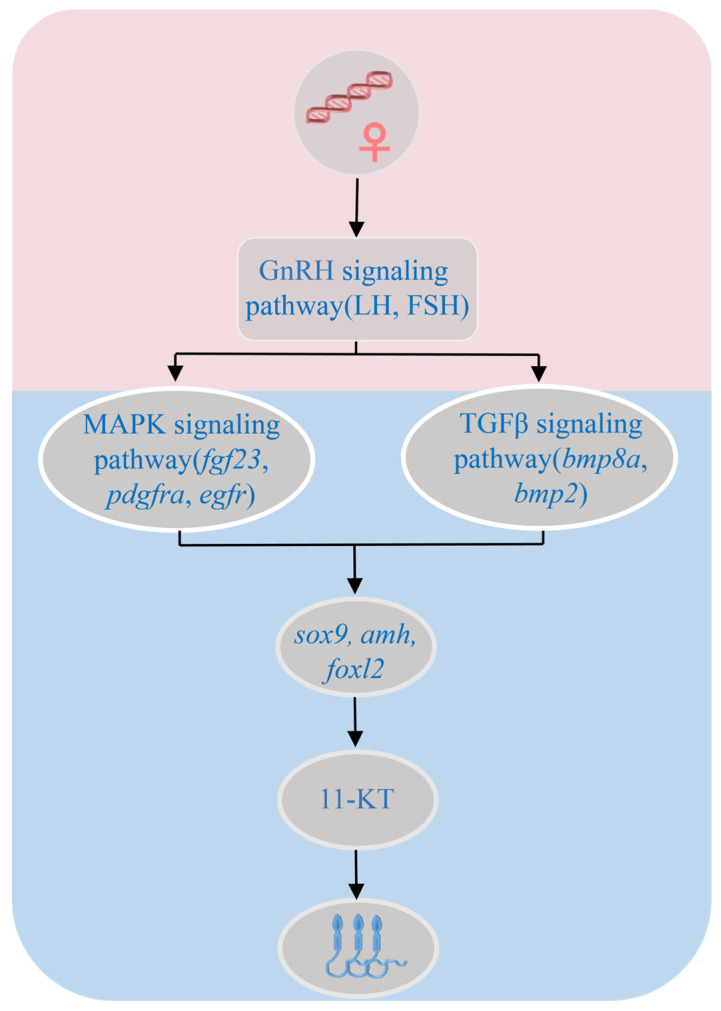
Hypothetical model of GnRH receptor antagonism-induced masculinization in genetic female *S. schlegelii* based on transcriptomic data. The proposed relationships are correlative, not causal, and require functional validation.

It should be emphasized that this study primarily employed transcriptome sequencing and gene set enrichment analysis to investigate changes in the gene expression profile following Relugolix treatment. We propose the following hypothesis: the GnRH signaling pathway may regulate the expression of key genes involved in sex differentiation and local gonadal steroidogenesis through the TGF-β and MAPK signaling pathways, thereby participating in the regulatory process of sex differentiation (Figure 7). However, this study lacks functional validation experimental evidence for these genes. The cellular localization of *bmp2* and *bmp8a* during gonadal development in *S. schlegelii* remains unclear; the causal relationship between the downregulation of *pdgfra* and *egfr* and the reduction in ovarian somatic cell numbers is merely speculative, and has not been confirmed through experiments involving cell proliferation, apoptosis marker detection, and somatic cell counting. This hypothesis remains to be further verified by subsequent functional experiments. Additionally, although three biological replicates were set for each group in this study—meeting the basic requirements for transcriptome sequencing analysis—this still presents limitations in reflecting biological variation. Increasing the number of biological replicates would more accurately represent biological differences among samples. Due to current constraints, this study was unable to include additional replicate samples. Follow-up studies with expanded sample sizes are needed, combined with techniques such as in situ hybridization and immunohistochemistry, to systematically validate the roles of these genes in sex differentiation of *S. schlegelii*, thereby further elucidating the molecular mechanisms underlying the GnRH-mediated temperature-dependent sex determination regulatory network.

## 5. Conclusions

In this study, transcriptome analysis combined with qRT-PCR verification clarified the regulatory role of GnRH signaling pathway in sex differentiation of *S*. *schlegelii*: GnRH signaling pathway can precisely mediate sex differentiation by regulating key genes associated with the TGF-β signaling pathway, MAPK signaling pathway, and steroid biosynthesis, and the expression changes in these key genes are somewhat correlated with gonadal development. Meanwhile, this study has certain limitations. Further functional experiments are needed to verify the specific roles and regulatory networks of individual genes, so as to improve the molecular mechanism of GnRH-mediated sex differentiation and provide theoretical support and experimental evidence for research on fish sex regulation and the optimization of monosex breeding techniques.

## Figures and Tables

**Table 1:** Transcriptome sequencing data of *S. schlegelii* in the control and experimental groups.

Sample	Raw Reads (×10^7^)	Clean Reads (×10^7^)	Q20 (%)	Q30 (%)	GC (%)
C1	4.955	4.935	98.72	96.29%	47.67%
C2	4.8	4.78	98.78	96.48%	47.72%
C3	5.305	5.283	98.94	96.95%	50.79%
T1	5.45	5.428	98.85	96.7%	47.54%
T2	6.31	6.283	98.91	96.85%	47.43%
T3	4.82	4.8	98.93	96.91%	47.51%

## Data Availability

All generated data are presented in the article and Supplementary Materials.

## Supplementary Materials

The following supporting information can be downloaded at: https://www.mdpi.com/article/10.3390/biology15110857/s1, Table S1. Primers for qRT-PCR Analysis of Related Genes in *S. schlegelii*; Table S2. PCR primers for genetic sex identification in *S. schlegelii*.
