# Supplementary material for: GnRH Regulates Sex Differentiation in Sebastes schlegelii Through TGF-β/MAPK Signaling Pathways
Source: Biology (Basel). 2026 May 30;15(11):857. doi: 10.3390/biology15110857 (PMC13255659; doi:10.3390/biology15110857)
Supplement: Supplementary file 1 [file biology-15-00857-s001.zip › biology-4266367-supplementary.pdf]

Supplementary Table S1. Primers for qRT-PCR Analysis of Related Genes in *S. schlegelii*

Gene	Forward primer5′ –3′	Reverse primer 5′ –3′	amplification efficiency
btf3l4	GAAGTTGGCCGTCAACAATA	GACAGAGAGGCTTGAACTTTG	103%
bmp8a	GCATGAACGCCACTAACCA	ATGCCTTTGGAACCTCGTCA	106%
bmp2	GGGGTGGAACGAGTGGATAG	CAGGCTCTGGGGATGTTTGA	98%
fgf23	ACCCGCACAACGTCTACTAC	GACACGTTGCGTCCATCATC	99%
pdgfra	CTACGGCATACTGCTCTGGG	ATGTAGGCGTCGTCGTTCTC	89%
egfr	CAGACGACACAGAGACGGAG	GGGTCTTGAAGGTGGGAGTG	71%
srd5	ATGCACCAATCAGCCTAACA	GGTGGGTCTGAGTTTTGTT	97%

Supplementary Table S2. PCR primers for genetic sex identification in *S. schlegelii*

Gene	Forward primer5′ –3′	Reverse primer 5′ –3′
sex marker	GTAAACCAAGAACTGAGGAGG	GAGAAAGCAGAAGTGGAATCA
